# Identification and characterization of allergens in spices by mass spectrometry

**DOI:** 10.1186/2045-7022-4-S2-P1

**Published:** 2014-03-17

**Authors:** Marlene Hummel, Tina Wigger, Jens Brockmeyer

**Affiliations:** 1 Westfaelische Wilhelms-Universitaet, Institute of Food Chemistry, Muenster, Germany

## Background

One of the major sources of hidden allergens in food is the use of spices that contain undeclared allergenic ingredients. Sesame and mustard are highly relevant in this context, as both seeds are frequently used in a variety of spices and can elicit severe allergenic reactions. Major allergens of mustard and sesame belong to the 2S-albumin family of seed storage proteins. 2S-albumins are members of the prolamin superfamily and are widely distributed in seeds and tree nuts. 2S-albumins are synthesized as a single precursor protein with about 13 kDa size that is subjected to extensive posttranslational proteolytic processing. The mature 2S albumins are composed of two subunits of approximately 9-10 kDa and 3-4 kDa size, respectively, which are linked by disulfide bridges. The 2S albumins are encoded by a multigene family possibly leading to numerous isoforms that may show considerable differences in their structures and allergenicity. The precise number, amount and characteristics of 2S albumin isoforms present in mustard and sesame however still remain unclear. One characteristic modification found is the C-terminal clipping of the small subunit that has been exemplarily described for Ses i 1 [1].

## Method

To analyze structural characteristics of 2 S albumin isoforms in detail, mustard and sesame seeds were ground to a fine powder in a nitrogen-cooled mill and defatted by pentane extraction. The dried powder was suspended in different buffers for 2 hours under continuous shaking. The supernatant was subjected to gel permeation chromatography on a preparative Superdex 200 column and equilibrated in 0.1 mM Tris-HCl (pH 8.0). Gel permeation fractions were applied to either a protein (Accucore 150-C4) or - after tryptic digestion - a peptide (Accucore 150-C18) column and analyzed by HRMS (high resolution mass spectrometry).

## Results

The major 2S-albumins of mustard (Sin a 1, Sin a 2) and sesame (Ses i 1, Ses i 2) could be purified by gel permeation chromatography and characterized using a combination of bottom-up and top-down proteomics. Using this appraoch, novel isoforms of 2S albumins in mustard could be characterized. In addition, unknown C-terminal clipping of the large subunit of 2S albumins in sesame was observed.

## References

[B1] Moreno FJ Biochim Biophys Acta 2005 1752 142 53 10.1016/j.bbapap.2005.07.022 16140598

